# Evaluation of a simple *Theileria annulata* culture protocol from experimentally infected bovine whole blood

**DOI:** 10.1051/parasite/2012193281

**Published:** 2012-08-15

**Authors:** M. Gharbi, R. Latrach, L. Sassi, M.A. Darghouth

**Affiliations:** 1 Laboratoire de Parasitologie, Université de la Manouba, École Nationale de Médecine Vétérinaire 2020 Sidi Thabet Tunisie

**Keywords:** *Theileria annulata*, cell culture, blood, Ficoll, *Theileria annulata*, culture cellulaire, sang, Ficoll

## Abstract

We have evaluated a new simple technique using whole blood from experimentally infected cattle for the isolation and cultivation of *Theileria annulata*. The study was carried out on 20 Holstein-Frisian bovines that had been experimentally infected with a virulent lethal dose of *Theileria annulata*. This technique has been compared to the classical peripheral blood monocyte isolation with Ficoll carried out on 22 experimentally infected Holstein-Friesian calves. The effectiveness of the reference technique was estimated to 86.4%, whilst the effectiveness of the new technique was 100%. Moreover, this new technique leads to time and money saving estimated to € 3.06 per sample. It decreases the contamination risks by reducing the steps of sample manipulation.

## Abstract (fr)

Les auteurs évaluent une nouvelle technique, très simple, permettant l’isolement de *Theileria annulata* à partir du sang total de bovins infectés expérimentalement. Cette étude a été réalisée sur 20 bovins de race Frisonne-Holstein qui ont été infectés par un stabilat létal de *Theileria annulata*. Cette technique a été comparée avec la technique conventionnelle d’isolement des monocytes avec le Ficoll. L’efficacité de la technique de référence a été estimée à 86,4 %, celle de la nouvelle technique est de 100 %. De plus, cette technique permet d’économiser du temps et de l’argent (3,06 *€*) par échantillon. Cette nouvelle technique diminue les risques de contamination en réduisant le nombre d’étapes de manipulation des échantillons.

Bovine tropical theileriosis (*Theileria annulata* infection) is a tick-borne disease that has been described in three continents: Europe, Asia, and Africa. *T. annulata* infection represents a major constraint to pure bred cattle development in endemic countries. The control of this disease can be carried out by the implementation of one or more of the three control options: (i) acaricide application on cattle and on the barn walls, (ii) roughcasting of the outer and inner wall surfaces of the barn, inducing destruction of the nymphs lodgings, (iii) vaccination with local attenuated cell line vaccine (Brown, 1990; Darghouth *et al.*, 2003). The development of a simple and low cost *T. annulata* culture technique is useful in several fields: epidemiology, immunology, molecular biology and vaccinology. The aim of the present study was to evaluate the effectiveness of a rapid and simple technique for *in vitro T. annulata* isolation from infected animal blood and to compare this protocol to the classical parasite culture technique based on isolation of peripheral blood monocytes by Ficoll gradients (Pipano *et al.*, 1990).

## Material and Methods

In order to compare the two techniques we used two groups of animals. The animals were purchased from a tropical theileriosis and *Hyalomma detritum detritum* free farm and they were kept in a tick-free isolation unit at the national veterinary school at Sidi Thabet:

- Group 1: 20 Holstein-Friesian bovines aged between five and ten months.
- Group 2: 22 Holstein Friesian calves aged between three and six months.

The animals were infected by subcutaneous injection of a lethal dose of 1.8 and 0.25 equivalent ticks using ground up tick supernates (GUTS) of the Jdaida 4 (Ta3/2 isolate), respectively.

Ficoll (Histopaque^©^ 1077, Sigma) was used for the isolation of peripheral blood monocytes (PBM), as recommended by the supplier. This technique is considered as the reference technique.

Culture media were prepared according to the protocol described in Brown (1981). Briefly, sterile RPMI-1640 (Gibco Invitrogen Australia Ltd, Victoria, Australia) was mixed with Heparin (Sigma) at the final concentration of 100 IU/ml, 2-Mercaptoethanol (Sigma) (10^-5^ M) and penicillin-streptomycin at the concentrations of 10^5^ IU/ml and 0.1 g/ml, respectively. The media was filtrated through a 0.2 µm Millipore^®^ filters and mixed with 10 % foetal calf serum (Gibco Invitrogen Australia Ltd, Victoria, Australia) at the concentration and 100 µM/ml L-Glutamine (Gibco Invitrogen Australia Ltd, Victoria, Australia).

Around day 12 post-infection, the animals of both groups presented typical clinical signs of tropical theileriosis. Blood samples were taken at the onset of clinical signs consistent with the onset of an episode of acute tropical theileriosis. Whole blood was aseptically collected in heparinised tubes (Vacutainer^©^).

For Ficoll (Histopaque^©^ 1077, Sigma) PBM isolation, ten millilitres of blood were aseptically collected in heparinised sterile tubes (Vacutainer^©^), peripheral blood monocytes were isolated as recommended by the supplier. For the whole blood technique only one millilitre of blood was mixed with complete RPMI-1640 (Gibco Invitrogen Australia Ltd, Victoria, Australia) in a 25 cm^3^ cell culture vial in vertical position. All cell cultures were incubated at 37 °C, 90 % humidity and 5 % CO_2_ for two weeks.

The cell cultures were monitored by making cytospin smears three times a week. The Giemsa stained cell culture smears were examined microscopically for *T. annulata* schizont infected cells. To compare the two techniques, six indicators were estimated:

1. Cell culture effectiveness is the number of positive cell cultures at the end of the survey divided by the number of starting cells.
2. Range of first day of passage is the minimum delay to obtain a schizont index (SI) superior to 50 %. SI is the ratio of infected WBC/examined WBC.
3. Number of contaminated vials.
4. Schizont index at day 9.
5. First-day of schizont detection.
6. Costs difference = cost of reference technique - cost of the rapid method (the labour costs and laboratory equipments were ignored).

## Results and Discussion

Compared to the reference technique, the rapid protocol is more effective, saving both time and money (Table I).

**Table I.** Comparison of two *Theileria annulata* isolation techniques: culture of whole blood and following Ficoll purification.

Indicator	Ficoll extraction	Whole blood
Cell culture sensitivity	19/22	20/20
Range of first day of passage	11 to 21	6 to 9
Number of contaminated vials	3/22	0/20
Schizont index at day 9 (in %)	ND	100
First day of schizont detection	ND	6
Costs difference (€)	Reference	– 3.06

ND: not done.

The control of tropical theileriosis is not possible until effective control policies are implemented, particularly vaccination with local attenuated cell line vaccines. To simulate the field conditions when the veterinarian will be called to examine clinically infected bovine, the animals were sampled at the beginning of the clinical signs corresponding to day 12 of infection.

Cells cultures were negative at day 0 and 3 indicating that the proportion of infected cells is low to zero. This can be attributed to the small quantity of stained samples when using the cytospin^®^ (30 µl per sample).

At day 6, the cultures showed a high proliferation rate, at day 9, the schizont index increases dramatically for all the animals, and reached 80 to 90 % at day 15. The index schizont progresses similarly to the conventional cell culture methods.

The parasite cultures isolated by Ficoll were successful for 19 out of 22 samples. Pipano *et al.* (1990) estimated the effectiveness of Ficoll isolation to 50%. However, they examined less severe clinical cases than those used in the present study. Indeed, they worked on animals challenged with *T. annulata* contaminated blood, whereas in our study the animals were challenged with the Jed 4 GUTS at the lethal dose of 1.8 to 2.5 tick equivalents. It has been established that infection with sporozoïtes induces a more severe infection than inoculation of *T. annulata* present in blood (Darghouth, 2000).

When considering the new rapid protocol, the day of first passage (first day when the schizont index is equal or higher than 50%) was estimated to either day 6 or day 9, whilst the first passage is realised from day 11 to 21 (median: day 15) for Ficoll. Cell culture from total blood allows a more rapid development of infected cells, since they are suspended in whole blood containing several growth factors leading a rapid growth of the cells and good output of cultures. Indeed, the *T. annulata*-infected cells can activate and induce non-specifically the proliferation of non-infected autologous T cells (Glass & Spooner, 1990), known as *Theileria* mixed lymphocyte reaction (*Theileria* MLR). In MLR, the non-specifically recruited lymphocytes produce cytokines that enhance the expansion of infected cells with a dramatic increase of INFγ (gamma interferon) and TNFα (tumour necrosis factor alpha) concentrations (Glass, 2001). These cytokines increases the proliferation of young schizonts and induce the production of activated macrophages that will secrete IL-2. During the early infection stage, exogenous IL-2 secreted by non-infected lymphocytes contributes to the initiation of infected cells transformation (Dobbelaere *et al.*, 1990).

The present technique offers a good environment to the cells, since they are not washed and centrifuged. Cell washing can lead to the loss of infected cells and decrease the output of cell cultures with an increase risk of contamination. Indeed, three out of 22 Ficoll treated samples were contaminated. One contamination was even observed before the first passage, whilst the two others were observed later.

The new technique leads to a dramatic saving in time, since it can be performed in few minutes, whereas the conventional technique requires two to three hours. In addition, the protocol saves € 3.06 per sample when compared to the conventional technique. Combined, they could encourage the use of this new technique for the isolation of *T. annulata* from infected animals and its validation under field conditions, where *T. annulata*-infected cattle are in a carrier state.
